# In-Situ Synthesis of Layered Double Hydroxide/Silica Aerogel Composite and Its Thermal Safety Characteristics

**DOI:** 10.3390/gels8090581

**Published:** 2022-09-13

**Authors:** Mengtian Sun, Yang Wang, Xiaowu Wang, Qiong Liu, Ming Li, Yury M. Shulga, Zhi Li

**Affiliations:** 1 School of Resources and Safety Engineering, Central South University, Changsha 410083, China; 2 Institute of Problems of Chemical Physics, Russian Academy of Sciences, Chernogolovka 142432, Russia; 3 National University of Science and Technology MISIS, Leninsky pr. 4, Moscow 119049, Russia

**Keywords:** silica aerogel, layered double hydroxide, thermal properties, structure

## Abstract

To adjust the thermal safety of hydrophobic silica aerogel, layered double hydroxide (LDH)/silica aerogel (SA) composites were prepared by an in-situ sol-gel process at ambient pressure. This study found the physical combination of SA and MgAl-LDH based on the FTIR spectra and phase composition of LDH/SA. The N_2_ sorption analysis confirms that the introduction of MgAl-LDH does not change the mesoporous attribution of LDH/SA significantly. With the increase in MgAl-LDH addictive content, the low density (0.12–0.13 g/cm^3^), low thermal conductivity (24.28–26.38 mW/m/K), and large specific surface area (730.7–903.7 m^2^g) of LDH/SA are still maintained, which can satisfy the requirements of thermal insulation. The TG-DSC analysis demonstrates that the endothermic effects and metal oxides formed during the MgAl-LDH decomposition are beneficial to the improvement of the thermal stability of LDH/SA composites. In addition, it was found that the gross calorific values of LDH/SA composites decrease with an increase in MgAl-LDH addictive content, all of which are lower than that of the pure SA. The research outcomes indicate that the thermal safety of LDH/SA composites is enhanced significantly by doping MgAl-LDH without impairing too many of the excellent properties, which benefits their expansion in the thermal insulation field.

## 1. Introduction

Silica aerogels (SAs) are a three-dimensional nanoporous material [1,2,3]. They have extremely low density (~3 kg/m^3^), low thermal conductivity (0.012~0.016 W∙(m∙K)^−1^), and high specific surface area (800~1200 m^2^/g) [2,4]. The excellent thermal insulation property of SAs allows for broad application prospects in the field of thermal insulation. However, the thermal insulation performance of SAs is significantly weakened in medium and high temperatures, especially when the temperature exceeds 300 °C [5]. The reason is that the hydrophobicity of SAs that are currently used as thermal insulation materials is lost at higher temperatures (200~400 °C), resulting in a decrease in the effect of thermal insulation and the service life of SiO_2_ aerogels [6]. At the same time, pure SAs have a weak ability to inhibit infrared radiation in the range of 100~1000 °C, and the radiative heat transfer of the aerogels will be significantly enhanced, which will lead to a definite increase in the thermal conductivity of SAs at high temperatures [7]. The three-dimensional porous framework of SAs will collapse as a result of the sintering behavior at high temperatures. This increases the amount of heat that is conducted in the solid phase, which will further weaken the thermal insulation properties of the aerogels. These greatly limit the application of SAs in the field of high-temperature thermal insulation. Therefore, there is an urgent need to develop a SiO_2_ aerogel composite material with excellent high-temperature thermal insulation properties to overcome the current shortcomings of SAs in the field of high-temperature thermal insulation.

Layered double hydroxides (LDHs) are a class of anionic lamellar compounds. They have drawn widespread attention due to their large specific surface area, anion exchange, and excellent thermal and mechanical properties and have been widely used in various fields such as catalysis [8], ion exchange [9], adsorption [10] and supercapacitor [11], etc. In particular, LDHs can act as flame-retardant nanofillers because of the characteristics of flame-retardant, halogen-free, non-volatile, and high-efficiency. Most commercial flame retardants release toxic gases during combustion, and some of these flame retardants incorporated into the polymer matrix may deteriorate the mechanical properties of the polymer. LDHs are a promising green flame-retardant material. They have a special layered structure and the advantages of inorganic flame retardants, such as magnesium hydroxide and aluminum hydroxide, which means LDHs play an important role in the field of flame retardants and have become a new generation of flame retardants and smoke suppressants in the application of polymers. Therefore, in recent years, polymer/LDH nanocomposites have received extensive attention due to their excellent and environmentally friendly flame-retardant properties. LDHs have been shown to effectively improve the thermal stability and flame retardant properties of many polymers, such as ethylene-vinyl acetate (EVA) [10], polyethylene (PE) [12], polypropylene (PP) [13,14], acrylonitrile-butadiene-styrene (ABS) [15], et cetera (see, for example [16,17,18]). Zhang et al. [19] fabricated a new LDH-based flame retardant (LDH-PCD) by a co-precipitation method, which enhanced the fire resistance and smoke-suppression properties of polypropylene (PP). Wang et al. [20] assembled layered double hydroxide nanosheet-silica (LDH-SiO_2_) nanocomposites in situ and used them to improve hydrogenated nitrile butadiene rubber (HNBR). The results show that the thermal stability and thermo-oxidative aging performance of LDH-SiO_2_/HNBR are better than those of SiO_2_/HNBR. Lu et al. [21] used a one-step hydrothermal method to directly grow MgAl-LDH on the inner surface of a wooden container, which greatly enhanced the flame retardant and smoke suppression properties. Few people add LDHs into silica aerogels to improve their thermal safety. Furthermore, the effects of LDHs on the properties of SA and the compatibility of LDH/SA composites have rarely been reported.

In this study, magnesium-aluminum layered double metal hydroxide (MgAl-LDH) was successfully prepared by a co-precipitation method, and MgAl-LDH was introduced into silica sol to prepare LDH/SA composites. We investigated the effects of MgAl-LDH doping with hydrophobic SAs on the microstructure, basic physicochemical properties, thermal stability, etc., of LDH/SA composites in detail. In addition, the effect of LDH doping content on the structure of LDH/SA composites was also investigated. Finally, this work confirms the feasibility of MgAl-LDH as a flame retardant to improve the thermal stability of hydrophobic SA. In the future, we will continue to study the flame-retardant properties and smoke suppression properties of composites with different contents of MgAl-LDH and reveal the corresponding flame-retardant mechanism.

## 2. Results and Discussion

### 2.1. Phase Composition of MgAl-LDH and LDH/SA Composites

**Figure 1:**
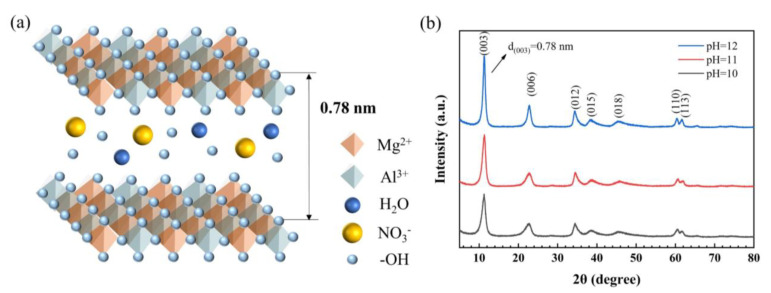
The schematic diagram (**a**) and XRD patterns (**b**) of MgAl-LDH prepared with various pH.

The schematic diagram of MgAl-LDH is shown in Figure 1a. The layered nanostructure MgAl-LDH consists of the positively charged metal hydroxide layers with intercalated anions (NO_3_^−^) and water molecules in the interlayer region, and the hydrogen bonds among these components help to stabilize the crystal structure of MgAl-LDH. Figure 1b shows XRD patterns of MgAl-LDH prepared at a pH of 10, 11, and 12. The characteristic diffraction peaks of MgAl-LDH are found at 2θ of 11.34°, 22.75°, 34.40°, and 38.50°, which corresponds to the reflections of (003), (006), (012), and (015) planes, respectively [22]. All these characteristic diffraction peaks are consistent with those of standard layered double hydroxide [23], indicating the prepared MgAl-LDH shares a typical layered nanostructure. A substrate spacing exists between the substrates of MgAl-LDH due to the intercalation of water molecules and anions in the interlayer region. For MgAl-LDH, the crystal plane (003) has a 2θ angle of 11.34°, and the substrate spacing *d*_(003)_ of the prepared MgAl-LDH is confirmed as 0.78 nm, according to the Bragg formula [24].

(1) $$d_{\left(003\right)}=\frac{n\lambda }{2\sin\theta }=\frac{1\times 0.15418}{2\sin(11.34/2)}=0.78$$

We also find that the as-prepared MgAl-LDH has high purity as there is no other peak observed in the XRD pattern except for the characteristic diffraction peaks derived from MgAl-LDH. In addition, it finds that the intensity of the diffraction peak of MgAl-LDH enhances with the increasing pH, which indicates a higher crystallization degree. In general, highly crystalline samples generally have better stability. It is necessary to add silica aerogels to MgAl-LDH with high crystallinity. In the following content, all the MgA-LDH were prepared at a pH of 12 and used as a dopant to synthesize LDH/SA composites.

**Figure 2:**
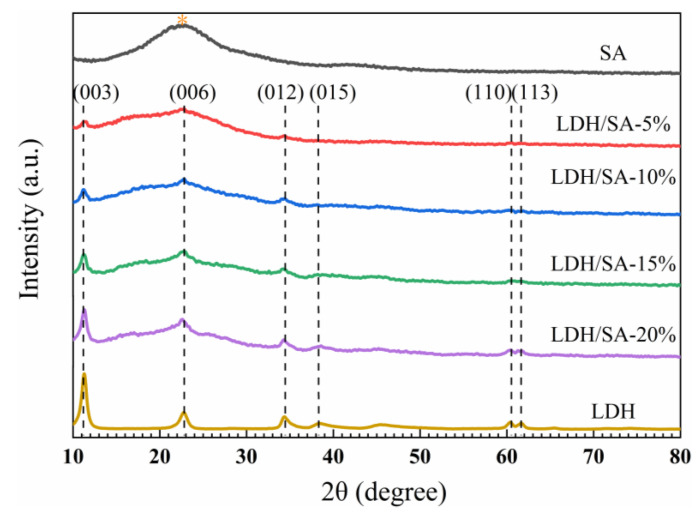
XRD patterns of the pure SA, MgAl-LDH and LDH/SA composites.

Figure 2 compares the XRD curves of the pure SA, MgAl-LDH and LDH/SA. From the XRD pattern of the pure SA, a wide peak at a low diffraction angle of 2θ = 23° is observed as an indication of the amorphous structure of the SA. The diffraction peaks of (003), (006) and (012) belonging to the layered structure of MgAl-LDH appear in the XRD pattern of the LDH/SA composite, which indicates that the sol-gel preparation process does not influence the formation and structure of the MgAl-LDH. For the LDH/SA, the XRD pattern can be regarded as the combination of the patterns of SA and MgAl-LDH, i.e., the superposition of the two. Furthermore, with the MgAl-LDH additive content increasing, the amorphous peak of SA is gradually impaired, while the intensity of the crystalline peak of MgAl-LDH obviously increases in the LDH/SA composites. Therefore, the XRD patterns of the LDH/SA seriously depend on the relative content of the SA and MgAl-LDH, in which the two components exist independently in the LDH/SA composites.

### 2.2. Microstructures of MgAl-LDH and LDH/SA

**Figure 3:**
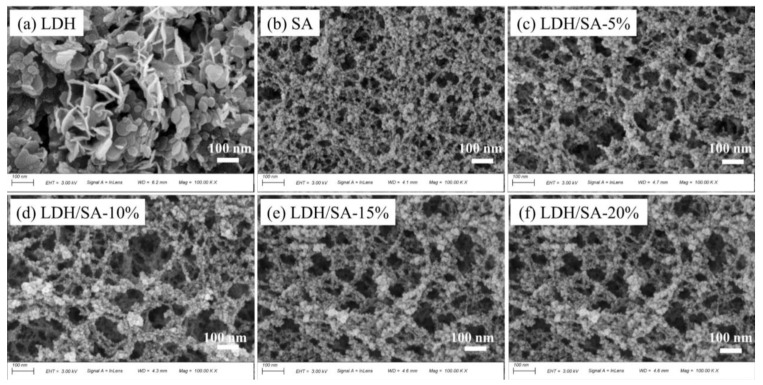
SEM images of MgAl-LDH (**a**), SA (**b**), and LDH/SA-5%~20% (**c**–**f**).

**Figure 4:**
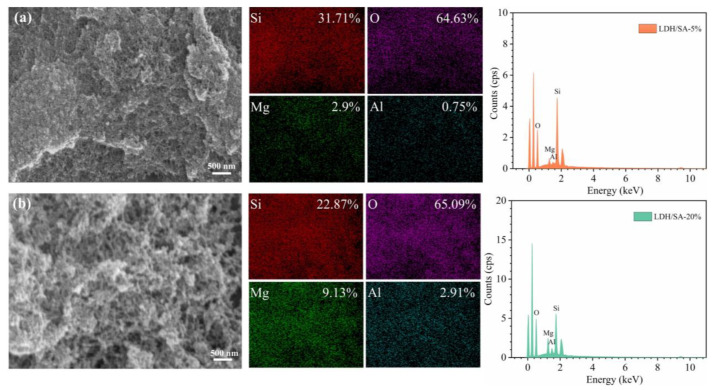
EDS spectra of the LDH/SA-5% (**a**) and LDH/SA-20% (**b**).

Figure 3 shows the microstructures of pure LDH, SA and LDH/SA composites containing 5%, 10% and 20% of MgAl-LDH, respectively. In Figure 3a, MgAl-LDH is made up of aggregated irregular nanosheets, which is a typical hydrotalcite morphology, as reported in Ref. [25]. However, the high charge density of the LDHs layers and the high content of anionic species and water molecules result in strong interlayer electrostatic interactions between the sheets, which lead to a tight stacking of the lamellae, resulting in the agglomeration of LDH nanosheets. The pure SA and LDH/SA composites all present a classical 3D nanoporous network in Figure 3b–f. It can be seen in Figure 4 that both pure SA and LDH/SA composites are porous materials. SEM images can qualitatively judge the morphology and pore size of pure SA and LDH/SA composites. The pore size of pure SA is mostly mesoporous, and it is obvious that the pore size is less than 100nm in Figure 3b. Furthermore, the pore size of all the LDH/SA (>100 nm) is obviously larger than that of the pure SA, and the pore size of the LDH/SA seems to increase with the LDH additive content in Figure 3c–f. We believe that the introduction of LDH leads to the collapse of the porous network of the aerogel, which increases the pore size. In regard to the combination between the LDH and SA, we speculate that the LDH nanosheets are wrapped by silica particles and aggregation, which means that they cannot be seen clearly. The detailed study on the pore structure and the combination between the two will be clarified further in the following context.

To further determine whether MgAl-LDH is doped into LDH/SA composites, the element distributions of the LDH/SA composites have been analyzed by the energy spectrum test (EDS), as shown in Figure 4. The silicon (Si) element comes from the silica skeleton network of SA. The magnesium (Mg) and aluminum (Al) elements derive from the metal hydroxide layers of MgAl-LDH. Both the SA and MgAl-LDH contribute to the oxygen (O) element. As seen from the EDS spectra, the Si, Mg, Al and O elements all distribute uniformly, and the distribution region of these four elements overlap each other. It indicates that the silica network is accompanied by the nanosheets derived from MgAl-LDH, though they are hard to find. Comparing the proportions of Mg and Al elements, we also find the atomic ratio of Mg and Al is close to 3:1, which is consistent with the initial preset atomic ratio in the synthesis.

### 2.3. Pore Structure

**Figure 5:**
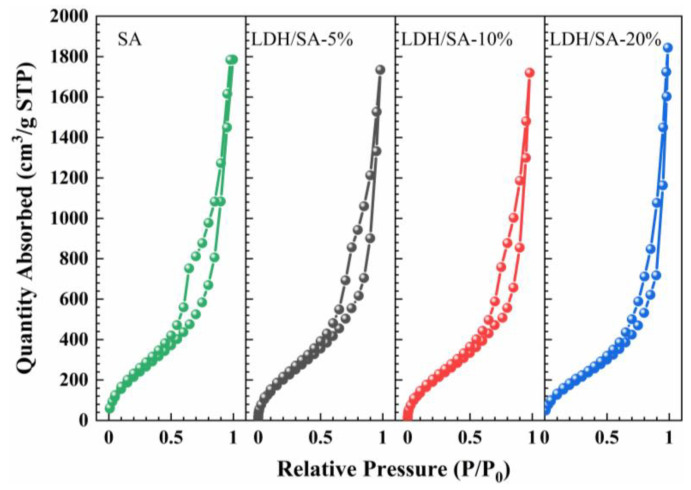
N_2_ adsorption–desorption isotherms of SA and LDH/SA composites.

The N_2_ adsorption–desorption isotherms are used to investigate the pore structure further. It can be seen that SA and LDH/SA composites have similar N_2_ adsorption–desorption isotherms in Figure 5. According to the IUPAC classification [26], all these N_2_ adsorption–desorption isotherms conform to the type IV isotherm, corresponding to the characteristic of mesoporous materials. The hysteresis loops of type H3 indicate the presence of slit-like interparticle pores [27]. Hence, the introduction of MgAl-LDH does not change the mesoporous attribution of the LDH/SA composites significantly.

The Barrett–Joyner–Halenda (BJH) method used to measure the pore size distribution has some limitations [28,29]. The BJH method is characterized in the range of 1.7–300 nm and is suitable for mesoporous materials of 2–50 nm. We believe that the introduction of LDH leads to the collapse of the porous network of the aerogel, which increases the pore size. The macropores of LDH/SA composites are difficult to detect by the BJH method. To determine the presence of macropores, we further calculated the total pore volume and the average pore size. The average pore diameter ($D_{pore}$) was calculated from the pore volume ($V_{pore}$) and $S_{BET}$ according to, $D_{pore}=4V_{pore}/S_{BET}$ where is derived from the bulk and skeletal density according to $V_{pore}=1/\rho_{b}-1/\rho_{s}$. Compared with $D_{pore}$ of LDH/SA-20% and SA, LDH/SA-20% increased significantly. This indicates that the introduction of LDH does lead to the collapse of the skeleton of LDH/SA, which decreases the specific surface area and increases the pore size. Moreover, $D_{pore}$ is greater than the average pore size*, indicating that many macropores exits.

The pore parameters, including specific surface area, total pore volume and average pore size are calculated and listed in Table 1. The BET-specific surface areas of all the three LDH/SA composites are smaller than that of the pure SA. With the MgAl-LDH additive content increasing, the BET-specific surface areas of the LDH/SA composites show an obvious downward trend, from 864.3 to 730.7 m^2^/g. The reduction in the specific surface area of the LDH/SA composites should be ascribed to the formation of larger clusters, which are derived from the aggregation of MgAl-LDH nanosheets and SA particles. Simultaneously, it is also accompanied by the collapse of the silica skeleton network during the ambient drying. As a consequence, the large pores (>100 nm), as shown in Figure 3c–e, are formed, and the average pore sizes of the LDH/SA composites increase significantly with the MgAl-LDH additive content.

### 2.4. Density, Porosity and Thermal Conductivity

**Figure 6:**
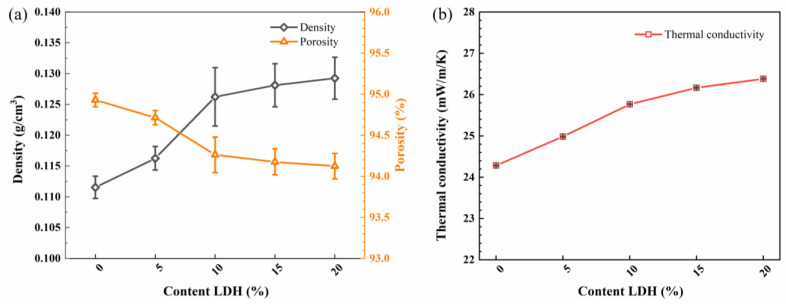
Density and porosity (**a**) and thermal conductivity (**b**) of SA and LDH/SA composites.

Figure 6 presents the variations of density, porosity, and thermal conductivity of the LDH/SA composites with the various MgAl-LDH additive contents. In Figure 6a, the density of the LDH/SA composites increases monotonously, which is caused by the increasing MgAl-LDH additive content. The corresponding porosity presents an opposite trend to the density, but the minimum porosity of the LDH/SA-20% is still higher than 94%. The increase in the density is attributed to the introduction of the MgAl-LDH since it has an obvious greater density than the pure SA.

Thermal conductivity is an important parameter to characterize the thermal insulation performance of materials. It can be observed that with the MgAl-LDH additive content increasing, the thermal conductivity of the LDH/SA composites also rises significantly from 24.28 to 26.38 mW/m/K. Because more heat transfer passageways are provided due to the addition of MgAl-LDH, more heat flows through the layered structure of MgAl-LDH rather than the nanoporous silica skeletons of SA. Consequently, the thermal conductivity of the MgAl-LDH rises. Note that even with the largest addition of 20% MgAl-LDH, the thermal conductivity of the LDH/SA composites only increases to 26.38 mW/m/K, which is still close to that of the still air (~26 mW/m/K at room temperature). Namely, with the MgAl-LDH additive content not exceeding 20%, the thermal conductivity of the prepared LDH/SA composites can still maintain an excellent level, indicating the nice thermal insulation performance.

### 2.5. Surface Properties and Hydrophobicity

**Figure 7:**
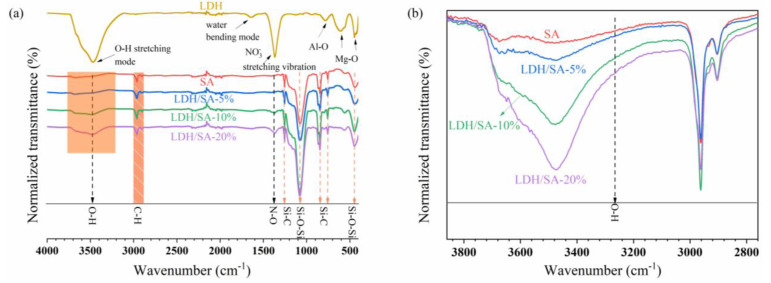
The FTIR spectra for the MgAl-LDH, SA, and LDH/SA composites (**a**) and the locally amplified IR spectra within the range of 2760–3860 cm^−1^ (**b**).

The FTIR spectrum further confirms the formation of MgAl-LDH by providing the characteristic peaks, corresponding to an interlayer anion, interlayer water molecules, an O-H of metal hydroxide layer and a Metal-O lattice in Figure 7a. In the infrared spectrum of the MgAl-LDH, the characteristic absorption band centered at 3466 cm^−1^ is attributed to the O-H stretching of the metal-hydroxide layer and interlayer water molecules [30]. The stretching vibration of N-O bonds from the interlayer anion, NO_3_^−^, appears at 1367 cm^−1^ [31]. The bending vibration of the water interlayer is presented at 1638 cm^−1^ [32]. The absorption bands observed at 410 and 780 cm^−1^ belong to the Al-O and Mg-O stretching vibrations, respectively [33]. For the IR spectrum of the SA, the symmetric and asymmetric stretching vibrations and the shear bending vibration of C-H bonds are observed around 2980–2880 cm^−1^ [34]. The peaks at 1260, 847 and 785 cm^−1^ belong to the Si-C bonds [35], indicating the presence of Si-(CH_3_)_3_ groups introduced by surface modification. These Si-(CH_3_)_3_ groups on the silica skeletons are the chemical basis for the hydrophobicity of SA.

Furthermore, it finds that the IR spectrum of the LDH/SA is the superposition of the IR spectra of the SA and MgAl-LDH, and the slight difference mainly lies in the peak intensity. For example, the peak of the N-O stretching vibration becomes more obvious with the MgAl-LDH additive content. Note that the peaks of Al-O and Mg-O from the MgAl-LDH are hard to observe, which may overlap with the Si-O from the pure SA. That is to say, there is no new chemical bond formed on the prepared LDH/SA composites. Therefore, the formation of the LDH/SA is considered the physical combination between SA and MgAl-LDH, which should be induced by electrostatic adsorption [36]. Figure 7b, also shows that the addition of LDH to pure SA leads to a noticeable increase in the peak intensity of the -OH groups, indicating a significant increase in the concentration of -OH groups. That inevitably influences the surface chemistry of the LDH/SA composites, e.g., hydrophobicity, which will be discussed in the following context.

**Figure 8:**
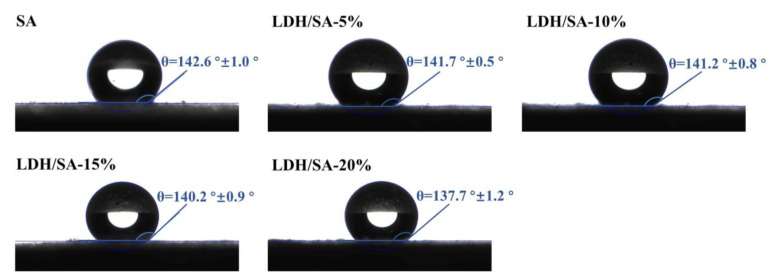
Contact angles of the pure SA and LDH/SA composites.

The contact angles of LDH/SA composites prepared with different MgAl-LDH additive contents are shown in Figure 8. The contact angle of the pure SA is around 143°, presenting good hydrophobicity. With the MgAl-LDH additive content rising to 20%, the contact angle gradually decreases to 138°, which suggests that the hydrophobicity of the LDH/SA composites is relatively impaired. As it is known to all, the hydrophobicity depends on the surface chemical composition [37] and roughness [38,39]. Considering that the contact angles were measured on the powdery sample, it can be assumed that all the tested samples have similar surface roughness. With a large number of -OH groups derived from the MgAl-LDH being introduced into the LDH/SA composites, more hydrophilic groups are distributed on the sample surface. As a consequence, the hydrophobicity of LDH/SA composites decreases with an increase in the MgAl-LDH additive content, which is consistent with the results drawn in Figure 8.

### 2.6. Thermal Safety Characteristics

#### 2.6.1. Composition Analysis of LDH/SA under Heat Treatment

**Figure 9:**
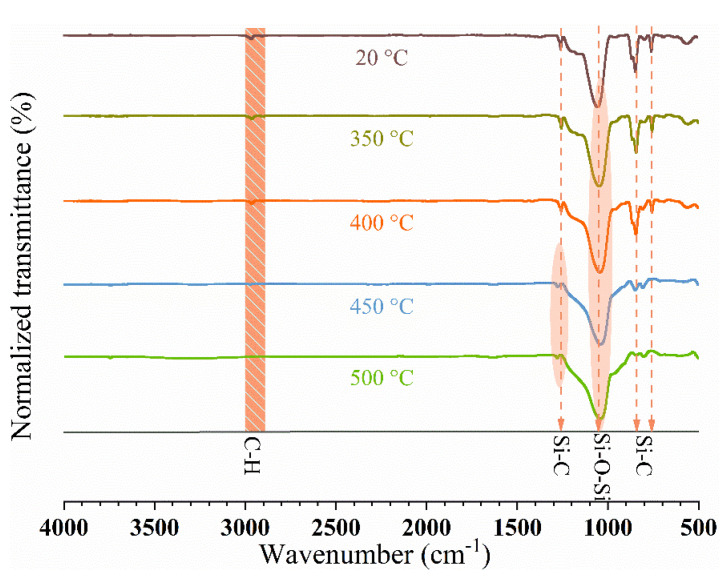
FTIR spectra of LDH/SA-10% by heat treatment at various temperatures.

Figure 9 shows the FTIR spectra of LDH/SA-10% under heat treatment at different temperatures. The primary absorption peaks of Si-O-Si (~1100 cm^−1^), Si-C (1260 cm^−1^, 847 cm^−1^ and 785 cm^−1^) and C-H (~2950 cm^−1^) have been analyzed in detail in the previous section. With the heat treatment temperature rising, a slight shift to the lower wavenumber of Si-O-Si bonds is observed, which should be ascribed to the effects of the dehydration reaction among Si-OH groups. When the heat treatment temperature exceeds 400 °C, the Si-C bonds at 1260 cm^−1^ slightly shift to the left larger wavenumber, 1277 cm^−1^, which indicates that the Si-(CH_3_)_3_ groups have transformed into Si-(CH_3_)_2_ groups under 450 °C, as reported in the Ref. [40]. In addition, it finds that the intensity of the Si-C bonds at 847 and 785 cm^−1^ become weaker and the same trend also occurs in the C-H bonds. All these changes in the Si-C and C-H bonds are related to the thermal decomposition of Si-(CH_3_)_3_ groups during the heat treatment.

**Figure 10:**
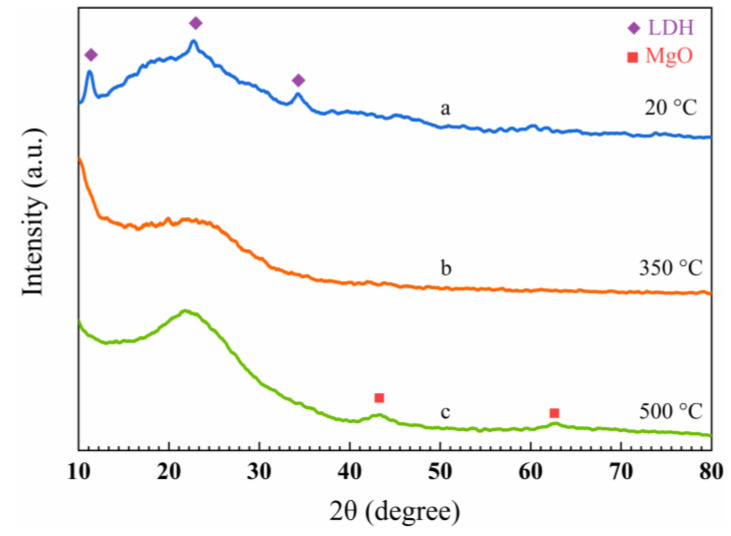
XRD patterns of the LDH/SA-10% by heat treatment at various temperatures, (**a**) the original, (**b**) 350 °C and (**c**) 500 °C.

It can be seen in Figure 10 that the three curves all have the typical wide peaks (2θ = 22°), which correspond to the amorphous diffraction peaks of silica aerogels. As the heat treatment temperature increases to 350 °C, the diffraction peaks at (003), (006), and (012) disappear, which suggests that the layered structure of the LDH/SA-10% has been destroyed in this condition. With the heat treatment temperature further rising to 500 °C, the MgO crystal diffraction peaks (2θ = 43.26°, 62.81°) appear in Figure 10c, implying that the LDH has decomposed to produce the MgO phase. Based on the obtained XRD patterns, it is speculated that the reason for no formation of the Al_2_O_3_ crystalline phase is related to the high dispersity of the Al^3+^ in the MgAl-LDH.

#### 2.6.2. Thermal Analysis

**Figure 11:**
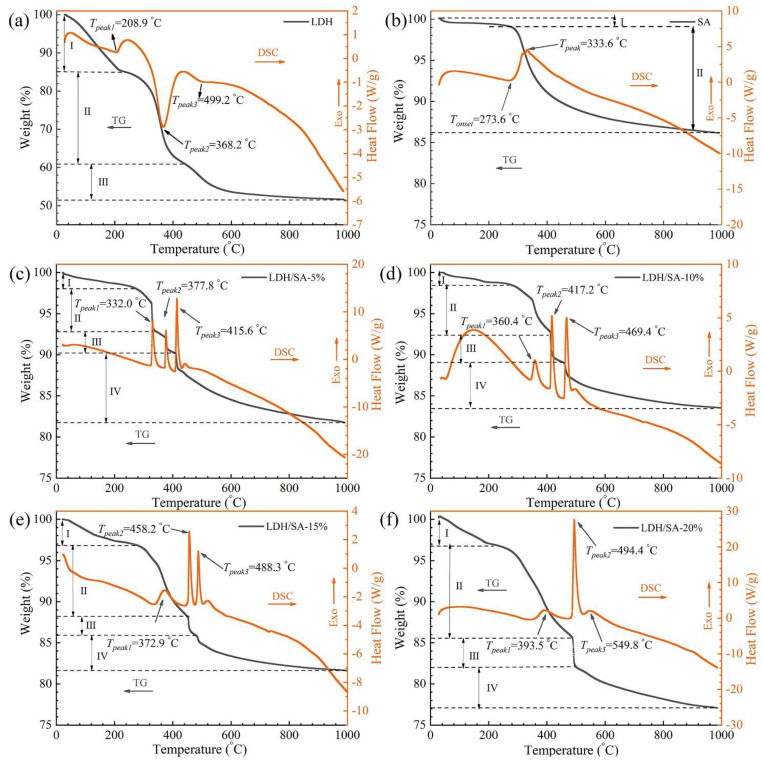
TG-DSC curves of (**a**) MgAl-LDH, (**b**) pure SA, and (**c**–**f**) LDH/SA composites under the heating rate of 10 °C/min in air.

Figure 11a shows the typical TG-DSC curve of the MgAl-LDH, which primarily undergoes three stages of decomposition. At Stage I (<220 °C), the weight loss is about 15%, which mainly corresponds to the removal of physically adsorbed surface water molecules and interlayer water [41]. In addition, this is an endothermic process, and the endothermic peak appears at 208.9 °C on the corresponding DSC curve. At Stage II (220~450 °C), the dehydroxylation occurs among the metal hydroxide layers on the laminate accompanied by the removal of anions [42], forming much water and leading to a 24% weight loss. Meanwhile, the dehydration reaction among the hydroxyls absorbs heat, resulting in the endothermic peak appearing at 368.2 °C on the DSC curve. At Stage III (450~600 °C), the weight loss is mainly caused by the removal of NO_3_^−^ anions between the interlayers [43], leading to a weight loss of 9.4%. The removal of NO_3_^−^ anions is considered related to their evaporation or decomposition, and the unobvious endothermic peak appears at 499.2 °C on the DSC curve. After the decomposition, the layered structure collapses completely, and the metal oxide, i.e., MgO, is formed finally.

Figure 11b presents the typical TG-DSC curves of hydrophobic SA, and the whole decomposition can be divided into two stages. The first stage is ascribed to the evaporation of residual solvent and water in the pores of the SA [40]. The second decomposition stage with the 13.3% weight loss begins at 273.6 °C, and the corresponding exothermic peak appears at 333.6 °C on the DSC curve. This process is mainly attributed to the thermal oxidative of Si-CH_3_ [44,45]. After this stage, the hydrophobicity of SA is transferred to the hydrophilicity, which implies the failure of SA while losing the ability to resist the water vapor in the air [45].

For the LDH/SA composites, the whole decomposition can be separated into four stages, as shown in Figure 11c–f. In Stage Ⅰ (<280 °C), the weight loss is caused by the evaporation of the residual organic solvent/water from the SA and the surface-adsorbed water and interlayer water from the MgAl-LDH. Stage II, III and IV are regarded as the combination of the thermal oxidation of Si-CH_3_ and the thermal decomposition of MgAl-LDH (including the dehydroxylation on the laminate and the removal of NO_3_^−^ anions between the interlayers). As discussed above, the thermal oxidation of Si-CH_3_ is an exothermic reaction, while the thermal decomposition of MgAl-LDH is an endothermic reaction. Obviously, the complementation occurs between the energy produced by the thermal oxidation of Si-CH_3_ and the one consumed by the thermal decomposition of MgAl-LDH. Considering the relative component content in the composition materials and the quantity of releasing or adsorbing energy, it is inferred that the energy released by the thermal oxidation of Si-CH_3_ covers the consumption by the thermal decomposition of MgAl-LDH. As a consequence, the broad exothermic peak of the Si-CH_3_ is cut into three relatively narrow exothermic peaks due to the complicated energy offset between the two. Simultaneously, each stage (II, III and IV) has a weight loss, which is also accompanied by a distinct exothermic peak.

From the above analysis, it can be seen that the thermal oxidative of Si-CH_3_ groups proceeds from Stage II to Stage IV; however, the decomposition of the MgAl-LDH just occurs in the midway. The important parameters, including the onset temperature (*T_onset_*) and peak temperature (*T_peak_*), can reflect the thermal stability and are listed in Figure 11 and Table 2. It finds the *T_onset_* of pure SA is 273.6 °C, while that of the LDH/SA increases from 327.2 to 367.7 °C, with the MgAl-LDH additive content increasing. The same increasing tendency also appears on the *T_peak_* of Stage II III and IV. All of these indicate that the thermal stability of the LDH/SA composite has been enhanced significantly. Based on the previous discussion, the endothermic reaction and the evaporation of the produced water adsorb much energy during the thermal decomposition of the MgAl-LDH. In addition, the finally formed metal oxides, e.g., MgO, can act as a high-temperature barrier material to resist heat transfer. All these are beneficial to delaying the thermal decomposition of the LDH/SA composites and finally lead to the improvement of their thermal stability.

#### 2.6.3. Gross Calorific Value

**Figure 12:**
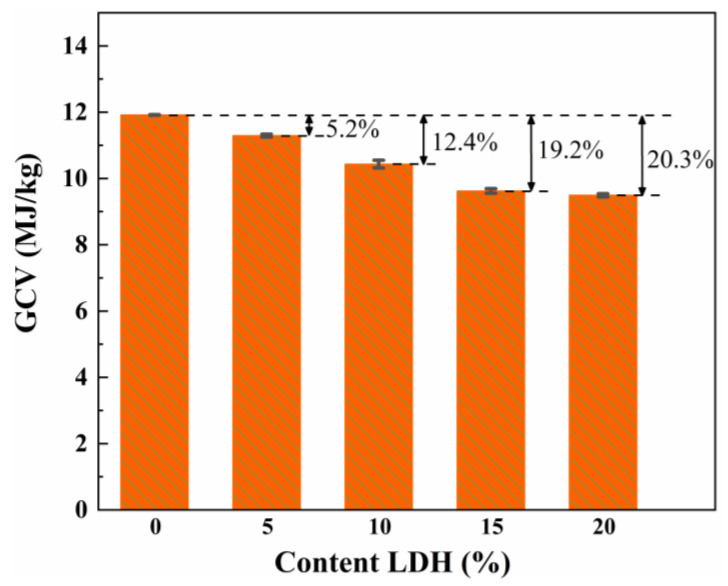
GCV of the pure SA and LDH/SA with various MgAl-LDH additive content.

Gross Calorific Value (GCV) reflects the total heat released by a material after it is completely burned [46]. As shown in Figure 12, the GCV of the LDH/SA composite decreases significantly with the increasing MgAl-LDH additive content. To be specific, the GCV of pure SA is 11.91 MJ/kg, while those of the LDH/SA composite drop from 11.3 to 9.493 MJ/kg. In the meantime, the descent rate of GCV increases from 5.2% to 20.3%, respectively. These data indicate that the introduction of MgAl-LDH reduces the GCV of SA, and higher thermal safety of the LDH/SA composites is achieved by this strategy.

## 3. Conclusions

In this work, the layered double hydroxide/silica aerogel (LDH/SA) composites were obtained by an in-situ synthesis. The microstructure, physicochemical properties and thermal safety characteristics of the LDH/SA were investigated in detail.

The study found that the microstructure of LDH/SA is similar to that of the pure SA without obvious change, and the formation of LDH/SA is deemed to be the physical combination of SA and MgAl-LDH. With the MgAl-LDH additive content increasing, the BET surface area and porosity both decrease, while the average pore size and thermal conductivity increase slightly. In spite of these, the lower thermal conductivity is still maintained (λ ≤ 26 mW/m/K), which indicates an excellent thermal insulation performance. Due to the endothermic effects and the formed metal oxides (e.g., MgO) during the thermal decomposition of MgAl-LDH, the thermal stability of the LDH/SA is improved significantly. The gross calorific value decreases obviously with the increasing MgAl-LDH additive content. Both of the two clearly imply that the as-prepared LDH/SA has an obvious enhanced thermal safety. In a word, this work demonstrates in detail that the in-situ synthesis of LDH/SA is feasible to enhance thermal safety without impairing too much of the thermal insulation performance, which provides an engineering example to further expand the application scenarios of pure hydrophobic SA.

## 4. Materials and Methods

### 4.1. Raw Materials

Al(NO_3_)_3_·9H_2_O (99.0%), Mg(NO_3_)_2_·6H_2_O (99.0%), Tetraethyl orthosilicate (TEOS), ethanol (EtOH, 99.7%), n-hexane (97.0%) were purchased from Sinopharm Chemical Reagent Co., Ltd. (Shanghai, China). Nitric acid (HNO_3_, 36–38%) and ammonia water (NH_3_·H_2_O, 25–28%) were used as the acid and basic catalyst, respectively. NaOH (96.0%) and Hexamethyl disilylamine (HMDZ, 99.0%) used in the experiments were purchased from Aladdin (Shanghai, China). Deionized water was made by an ultra-pure water machine (Direct-Q 3UV, Merck Millipore, Burlington, MA, USA).

### 4.2. Preparation of MgAl-LDH

First, 500 mL of deionized water was boiled, and N_2_ was passed into the three-necked flask for protection. Then, a certain amount of Mg(NO_3_)_2_·6H_2_O and Al(NO_3_)_3_·9H_2_O were weight and dissolved in 200 mL of deionized water. Next, the solution was transferred into a three-necked flask and heated to 72 °C under a water bath. A 0.8 mol/L NaOH solution was continuously dropped into a three-necked flask with constant temperature, and the pH in the three-necked flask was obviously to pH = 10~12. After that, the solution needs to stir vigorously for an hour to make Mg(NO_3_)_2_·6H_2_O and Al(NO_3_)_3_·9H_2_O fully react with NaOH to form the precipitation of magnesium-aluminum mixed metal hydroxide (MgAl-LDH). Finally, it was placed in a drying oven at 75 °C for crystallization for 24 h to obtain a milky white turbid liquid. The supernatant was removed, and the milky white turbid liquid was filtered and washed with distilled water several times until the pH of the supernatant was close to neutral. Finally, it was washed several times with ethanol to prepare LDH/EtOH emulsion (LDH/EtOH).

### 4.3. Preparation of LDH/SA Composites

**Figure 13:**
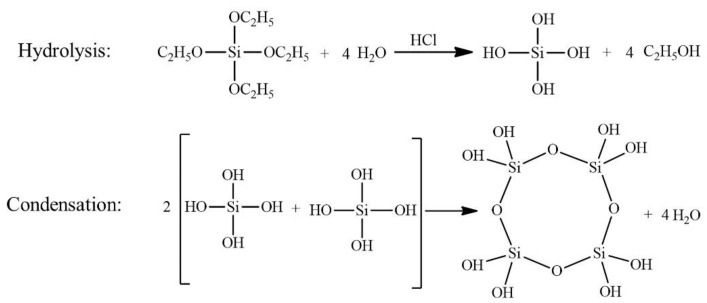
The reaction process of SA.

We choose TEOS as a silica precursor, EtOH as a solvent, water for hydrolyzing TEOS, and ammonia as a catalyst for gelation. The synthesis of LDH/SA composites involves four major steps: (1) the preparation of the silica sol; (2) dispersing LDH/EtOH in the silica sol; (3) preparing and modifying the alcogel; (4) drying the wet gels by ambient pressure drying. In step one, TEOS, EtOH, DI·H_2_O, and HNO_3_ were mixed with stirring for 5 min in a beaker and fully hydrolyzed in a 45 ^°^C water bath for 12 h. The reaction process includes a hydrolysis reaction and condensation reaction, as shown in Figure 13. In step two, NH_3_·H_2_O was added into hydrolysate and stirred for 5 min. The molar ratio of TEOS:EtOH:H_2_O:HNO_3_:NH_3_·H_2_O was fixed at 1:9.6:2.16:1.6 × 10^−3^:9.7 × 10^−3^.

**Figure 14:**
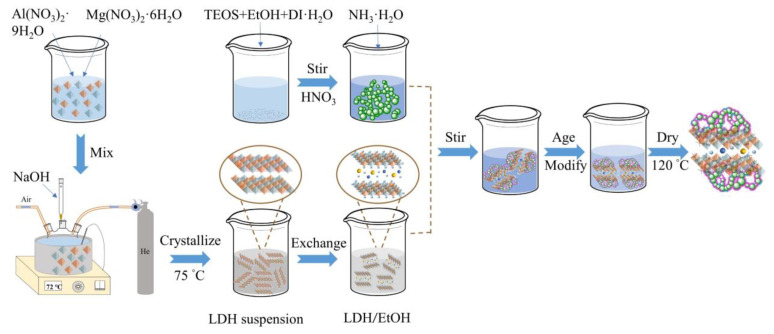
Flow chart of the process for synthesizing LDH/SA composites.

Then, immediately different contents of LDH/EtOH were dispersed in silica sol by mechanical stirring and ultrasonic treating (accounting for 0, 5, 10, 15, and 20% of total silica aerogel quality, and the samples denoted as SA, LDH/SA-5%, LDH/SA-10%, LDH/SA-15%, LDH/SA-20% respectively). The gelation generally occurred in 30 min. In step three, the generated LDH/alcogels were aged with EtOH for 12 h, exchanged with n-hexane for 12 h, and surface modified with HMDZ/n-hexane solution for 48 h, respectively. In step four, the wet LDH/alcogels were dried under ambient pressure at 120 °C for 4 h to obtain LDH/SA composites. The preparation process is shown in Figure 14.

### 4.4. Methods of Characterization

The success of the synthesis of MgAl-LDH and LDH/SA composites can be evaluated by characterization techniques such as SEM, EDS, XRD, FTIR, and thermogravimetric analysis.

The thermal conductivities of the composites were measured by a constant thermal analyzer (TC3000E, XIATECH, Xian, China) at room temperature. The amount of the sample for the thermal conductivity measurements is about 8–10 g, which was placed in two sample boxes. The pH was measured via a waterproof pen tester (7011, EZDO). The hydrophobicity was characterized by testing water contact angle. First, a 5 μL water droplet was dropped on the powdery sample surface, and the images were collected by the camera from a contact angle instrument (JC2000D1, Zhongchen Instrument, Shanghai, China). Whereafter, the contact angles were obtained through an image analysis by the ImageJ software. The tap density $\left(\rho_{t}\right)$ was measured by a tap density meter (ZS-202, Liaoning Instrument Research Institute, Liaoning, China) using a measuring cylinder of 10 mL with 300 r/min for continuous vibration within 10 min. The porosity was determined by Equation (2),

(2) $$Porosity=\left(1-\frac{\rho_{t}}{\rho_{s}}\right)\times 100\%$$

where $\rho_{s}$ is the skeletal density of SA (about 2.2 g/cm^3^).

The microstructure of pure SA and LDH/SA composites was observed by a field emission scanning electron microscope (SEM, Sigma 300, ZEISS, Oberkochen, Germany) accompanied by an energy dispersive spectroscopy (EDS, EDX-720, Shimadzu Corporation, Kyoto, Japan) to analyze the composition and content of elements of the MgAl-LDH and LDH/SA composites. The nitrogen adsorption-desorption isotherms were tested at 77 K using a QuadraSorb Station 2 analyzer (ASAP2020, Micromeritics, Atlanta, GA, USA). The Brunauer–Emmett–Teller (BET) method [47] was used to calculate the specific surface area, and the Barrett–Joyner–Halenda (BJH) method [48] was used to determine the pore size distribution, pore volume, average pore size, and other parameters. The pore composition of the samples was statistically analyzed, and the effect of the dopant characteristics on the porous structure of the hydrophobic SA was studied.

X-ray diffraction (XRD, D8 Advance, Bruker, Billerica, MA, USA) was used to determine the crystal phase of the specimens. The chemical bonds and chemical groups of pure SA, MgAl-LDH and LDH/SA composites were determined by a Fourier transform infrared spectroscopy (FTIR, Nicolet 8700, Nicolet, Madison, WI, USA) and the ATR method. The thermal stability analysis was tested using TG-DSC (SDT Q650, TA Instrument, New Castle, NH, USA), with a heating rate of 10 °C/min from room temperature to 1000 °C in air. The amount of SA and LDH/SA composites required for the TG-DSC test was about 5–10 mg.

The GCV of SA and LDH/SA composites were measured by using an oxygen bomb calorimeter. The amount was controlled at 0.3–0.4 g each time. Each sample was tested three times, and the combustion calorific value was obtained by calculating the mean value.

## Figures and Tables

**Table 1:** Physical properties of SA and LDH/SA composites.

Samples	BET Surface Area (m^2^/g)	Pore Volume (cm^3^/g)	Average Pore Size * (nm)	Total Pore Volume (*V_pore_*) (cm^3^/g)	Average Pore Diameter (*D_pore_*) (nm)
SA	903.7	2.8	12.2	8.5	37.6
LDH/SA-5%	864.3	2.7	12.4	8.1	37.7
LDH/SA-10%	814.5	2.7	13.1	7.5	36.7
LDH/SA-20%	730.7	2.9	15.6	7.3	39.9

*: The average pore size calculated by the BJH method includes micropores and mesopores.

**Table 2:** The detailed DSC data of pure SA and LDH/SA composites.

	*T_onset_* (°C)	*T_peak1_* (°C)	*T_peak2_* (°C)	*T_peak3_* (°C)
SA	273.6	333.6		
LDH/SA-5%	327.2	332.0	377.8	415.6
LDH/SA-10%	344.7	360.4	417.2	469.4
LDH/SA-15%	350.2	372.9	458.2	488.3
LDH/SA-20%	367.7	393.5	494.4	549.8

## Data Availability

Not applicable.
